# Predicting genetic evolution of viruses to identify suitable vaccines using artificial intelligence

**DOI:** 10.1038/s41598-026-35143-y

**Published:** 2026-02-03

**Authors:** Osama R. Shahin, Mohamed N. Ibrahim, Awadh Alanazi, Fahd S. Alharithi, Yasir Alruwaili, Ahmad A. Alzahrani, Eman Fawzy El Azab

**Affiliations:** 1 Department of Computer Science, College of Computer and Information Sciences, Jouf University, Sakaka, Saudi Arabia; 2 Department of Clinical Laboratories Sciences, College of Applied Medical Sciences at Al Qurayyat, Jouf University, Al Qurayyat, 77454 Saudi Arabia; 3 Department of Clinical Laboratory Sciences, College of Applied Medical Sciences, Jouf University, Sakaka, Saudi Arabia; 4 Department of Computer Science, College of Computers and Information Technology, Taif University, Taif, Saudi Arabia; 5 Center for Health Research and Innovations, Deanship of Graduate Studies and Scientific Research, Jouf University, Sakaka, Saudi Arabia; 6 Department of Computer Science and Artificial Intelligence, College of Computing, Umm-AlQura University, P.O.Box 8XH2+XVP, Mecca, 24382 Saudi Arabia

**Keywords:** Artificial intelligence, Genetic prediction, Genomic analysis machine learning, Vaccine development, Viral evolution, Computational biology and bioinformatics, Evolution

## Abstract

The evolution of the viruses is rapidly becoming a global challenge to the creation of vaccines since the new variants are often capable of escaping the immune system and decreasing the vaccine efficacy. The traditional methods of genomic epidemiology rely on the retrospective phylogenetic analysis, which can elucidate the previous mutations, but cannot predict the evolutionary trends in the future. In order to address these disadvantages, a new Refined Deep Evolutionary Learning Framework (R-DELF) is proposed that combines the genomic, structural, and temporal intelligence in predicting proactive viral mutations and assessing vaccine suitability. The methodology uses an ESM-2 Transformer that extracts structure-aware embeddings, merged with dual-attention Graph Neural Networks (GNNs) which learn phylogenetic and structural dependencies. Evolutionary learning maximiser improves adaptation modelling and an Explainable AI layer, which offers interpretability based on residue-level attribution. Tests indicate that experimentally it achieves 99.2% accuracy, 97.92% precision, 98.89% recall and 99.4% F1, which is higher than the current AI-based virology models. It is implemented in Python and with the help of TensorFlow and genomic and protein data obtained via Kaggle. The framework allows predicting the high-risk mutations in advance, facilitates the production of vaccines on time, and increases the preparedness to pandemics by making intelligent, data-driven predictions of viral evolution.

## Introduction

The rates of virus evolution are extremely high, and they are facilitated by the errors in replication, the pressure of the immune system, and cross-species transmission incidences^1^. These mutations have repeatedly been known to cause vaccines to be ineffective (partially or entirely) and were common occurrences throughout history, requiring re-formulation and booster drives. The COVID-19 pandemic has given a vivid example of this issue^2^: several variants of SARS-CoV-2 of concern (VOCs) were identified within months, and each had its unique properties of immunological escape^3^. The same type of evolution has been experienced with influenza viruses, HIV and other fast developing RNA pathogens^4^. Such constant shifts highlight the urgent necessity of more evolutionary, proactive approaches to biomedical challenges that will be able to predict the evolution of the virus and respond to it instead of reacting to it. Traditionally, the development of vaccines is based on the reactive model: researchers observe the strains present on the market, determine large new variants, and consequently change the composition of vaccines. Not only will this method take time but it is usually slow in tracking the evolutionary path of the virus^5^. The impact of delays in vaccine redesign in the event of global health crisis can be prolonged outbreak, strain on the health care system and other burdensome socio-economic effects^6^. There has been an improvement in genomic surveillance systems over the last few years to generate significant global sequence data, but it largely remains a surveillance tool, not a predictive engine. That is, data collection has been more ahead of data-driven foresight^7^. The possibility of Artificial intelligence (AI) has now provided such a solution to such a gap, where passive genomic archives can be transformed into proactive tools to predict future virus mutations before they become widely spread^8^. New possibilities to decode viral evolution at an unprecedented level of resolution have been developed in recent years by the new opportunities of biological deep learning, in the form of protein language models and graphical reasoning^9,10^. Nevertheless, regardless of their potential, the majority of AI-based virology systems are either predictive or interpretable, or biologically motivated. It requires a next-generation framework: a framework that does not toxically envision evolution, structure, and time of viral proteins but actually converts the results into practical vaccine targets^11^. Viral adaptation had already favourably changed the epidemiological situation before vaccines or therapeutics could be modified. Even in well-funded genomic surveillance networks, such as those in Europe and North America, the turnaround time from variant detection to vaccine update spans months. In regions with slower laboratory infrastructure, the lag is far greater.

### Limitations of traditional genomic epidemiology

The conventional genomic epidemiology has been based on the use of phylogenetic trees reconstruction, lineage tracing, and clade frequency to comprehend the evolution of viruses over time. Although these techniques have been very useful in retrospective surveillance^12^, they are essentially observational systems and not predictive ones. These methods have an inherent retrospective bias since they are based on historical mutation data: these methods can only reveal an outcome of evolution after it has already transpired in a circulating viral population^13^. This delay is especially concerning to highly mutating pathogens like SARS-CoV-2 and influenza, in which changes of antigenic interest can be generated with relative ease within evolutionary periods of short time^14^.

Conventional genomic methods are primarily interested in the analysis of the lineage, but not in the biochemical significance of single residues^15^. They do not consider variations of residues together, stability limitations and immune constraints which define mutation viability. These approaches ignore epistatic interactions that influence the adaptation of the virus by treating mutations as single events^16^. Without the presence of the time forecasting, they have the ability to explain evolution in the past but are unable to predict the occurrence of mutations in the future, and this leads to delays in response with the vaccine. Also, they do not think about structural feasibility some statistically viable mutations destabilize the protein folding or receptor binding and become biologically irrelevant^17^. In order to counter these constraints, predictive models should combine time dynamics, structural modeling, and evolutionary fitness. The hybrid method proposed in this study addresses this gap, which is the combination of deep sequence embeddings and graph-based structural argumentation and evolutionary learning to be able to proactively detect high-risk mutations before they become relevant at scale.

### Research motivation

Conventional genomic surveillance is reactive and detects mutations only after spread. AI enables proactive prediction of biologically meaningful viral evolution by modeling co-mutations, structural constraints, and immune escape. This supports early risk-site identification, anticipatory vaccine design, improved interpretability, ethical deployment, and strengthened pandemic preparedness for emerging pathogens.

### Key contributions

- The paper presents a proactive computational model that simulates the viral genetic evolution as a time-varying process to facilitate proactive knowledge of the mutation possibility of such a virus experiencing the actual immune and host forces.
- Integrates genomic, structural, and functional data layers to capture multi-scale biological dependencies, ensuring a holistic representation of viral adaptation mechanisms beyond sequence-level correlations.
- Embeds infer interpretability and evolutionary rationale in the core of the analysis of the model, which allows making a clear trace of the drivers of mutation and biological trade-offs on the viral fitness and immune escape.
- It expands AI applicability to emerging and under-sequenced pathogens by leveraging evolutionary constraints, enabling prediction capability even when genomic data is limited or incomplete.
- The paper gives ethical and governance principles of responsible AI utilization in virology, to resolve dual-use threats and guarantee the security of its application in global vaccine preparedness efforts.

The paper is structured in the following way; section-II summarizes related AI-based viral mutation studies; section III is the conceptual framework, section-IV is the methodology and model design, section-V is the implementation and evaluation and, discussion of findings and implications to proactive vaccine design and, section-VII is the conclusion with contributions, limitations and future directions.

## Literature review

The studies under review highlight the increasing use of AI in making predictions of the mutation of viruses via deep learning, biophysical simulations, and genomic data. Existing techniques are more effective in increasing the early detection and vaccine design, but there is a limitation in the quality of the data, interpretation, generalization, and the use with real-time surveillance systems.

Zou^18^ aims to improve prediction of future SARS-CoV-2 mutations by overcoming the limitations of applying conventional GPT models directly to noisy viral genomic sequences. It introduces PETRA, a pretrained evolutionary transformer that uses phylogenetic tree–derived evolutionary trajectories rather than raw RNA sequences to model viral evolution. PETRA is used to forecast emerging mutations, processing structured mutation pathways with weighted temporal and geographic sampling to address global sequence imbalance. Results show substantial gains over state-of-the-art baselines, achieving high recall for nucleotide and spike mutations and accurately predicting mutation patterns for major clades such as XEC and LP.8.1 before their global emergence. Limitations include PETRA’s inability to model recombination events, lack of prediction for phenotypic traits such as severity or immune escape, and persistent data imbalance from under-sampled regions.

Amran et al.^8^, uses a genomic analysis with a combination of artificial intelligence (AI) to forecast viral mutations and estimate risks of pandemics. The methodology implies the identification of genetic markers of virulence and transmissibility with the help of genomic sequencing, and the further analysis of genetic data with the help of modern machine learning algorithms to predict potential patterns of mutation under the conditions of replication rates, host interactions, and environmental factors. The limitations of the study however, are that it uses quality of data, it is difficult to model complex evolutionary dynamics and interdisciplinary cooperation is needed to make sure there is correct predictive models that are ethical and applicable worldwide.

Thadani et al.^19^, proposes EVEScape, a flexible and scalable system that combines deep learning, biophysical simulation, and structural modeling to forecast viral escape mutations during an initial pandemic. The method is a deep generative model which is trained on historic viral sequences and biological constraints based on protein structures and physical properties that can measure mutation fitness and escape potential. The framework has weaknesses such as the inability to capture new constraints in the immune or environmental responses that arise in the event of new pandemic and the dependence on past evolutionary information. Its predictive capability can be optimally met when it is combined with experimental validation and current pandemic statistics to additionally improve escape mutation predictions and improve global vaccine preparedness.

Tang et al.^20^, seeks to increase the level of preparedness against pandemics through the establishment and combination of computational techniques that will assess the probability of mutations in viruses, especially those that allow viruses to escape the vaccine. It uses two main methods forward mutation prediction, which is used to reconstruct phenotypes with respect to genotypes and reverse mutation prediction which uses deep mutational scanning (DMS), immunological profiling and machine learning to predict potential mutations before they occur. The approach is a combination of genome and phenotype-wide data to model the evolution pathways and immune escape. The findings support the idea that integrated prediction methods enhance effectiveness in predicting the evolution of viruses, which can be used in the design of proactive vaccines. Limitations however include the inability to consistently evaluate the metrics, dissimilar datasets used by the different models and difficulty in aligning predictive modeling and clinical and public health systems in real-time worldwide.

Bagabir et al.^21^, applies different AI methods, such as machine learning (ML), deep learning (DL) and artificial neural networks (ANN) to determine genomic sequences of SARS-CoV-2 and underpin the creation of drugs and vaccines to combat the COVID-19. The quality of the data and regional validation also influence the reliability of predictions, which makes it important to tackle the issue with the help of effective genomic surveillance and international cooperation to guarantee the precision, transparency, and efficiency of AI-based responses to pandemics. This is because Hamelin et al.^22^, seeks to predict the evolution of viruses by anticipating pathogenic mutations, before they happen, and thus results in proactive actions against the epidemic and pandemics by the public health authorities. It manipulates sophisticated artificial intelligence methods, most especially, deep learning and language models, in combination with genomic, epidemiologic, immunologic, and biological data. Its methodology implies training AI models by using large-scale data on viruses, including the ones obtained due to SARS-CoV-2, to detect patterns of mutations and predict evolutionary trends among RNA viruses. Nevertheless, the study has weaknesses such as lack of data, biases in sampling, and inability to simulate intricate evolutionary jumps or saltation occurrences, which makes it difficult to predict.

Sarmadi et al.^23^, also tries to discuss the uses of Artificial Intelligence (AI) to expedite and streamline the process of vaccines development. It uses a range of AI and machine learning (ML) models, e.g. Support Vector Machines (SVM), neural networks, and Recurrent Neural Networks (RNNs) to prioritize vaccine candidate proteins, predict binding scores, identify potential epitopes and design multi-epitope vaccines, as well as, track the viral RNA mutations. The approach entails the training of ML models using big biological and chemical data to acquire patterns to aid in the assessment of protein suitability and foresee molecular interactions. The result of the output indicates that AI has the ability to improve the accuracy or efficiency of vaccine design. The study has however, limitations in terms of dataset, computationalism and generalization to other viruses and mutation patterns.

The objective of Domingo et al.^24^, is to explain the biological consistency of mutation rates and error repair systems in the process of viral RNA replication and how they affect viral adaptability and genome stability. It uses experimental and theoretical studies of viral polymerase fidelity and evidence repairing processes, especially the 3’−5’ exonuclease present in coronaviruses. The methodology includes reviewing the measurements of the mutation rate, the dynamics of mutant spectrum, and the evolutionary models of the information maintenance. Yet, there are such constraints as the absence of a clear comprehension of the time dynamics of the mutant swarm development, limited information on the minority variants, and difficulties with the usage of consensus sequences in order to design universal vaccines or antivirals.

The objective of Doneva and Dimitrov^25^ is the construction of the correct machine learning models to predict protective viral immunogens so as to improve vaccine design. It employs some computational methods that involve the use of E-descriptors along with auto- and cross-covariance transformations to encode the protein structures into numeral vectors. Its methodology includes training and testing models with 1,588 immunogenic and 468 non-immunogenic viral proteins and feature selection was performed through the gain/ratio approach. Random Forest, Multilayer Perceptron, and XGBoost algorithms were applied, achieving superior predictive performance compared to the established VaxiJen 2.0 tool. However, limitations include dependence on available datasets, potential bias toward known viruses, and limited generalization to novel or less-studied viral strains.

Enhancing protein sequence-based modeling by introducing a pre-training strategy that captures short- and long-range co-evolutionary interactions often missed by traditional protein foundation models^26^. The technique is used to improve structural and functional prediction by learning residue-interaction patterns from large-scale sequence data. The method processes sequences through a co-evolution–aware pre-training framework that integrates interaction-focused loss functions. Results show superior generalization and performance over baselines, including ESM-2, highlighting stronger co-evolution feature extraction compared with approaches such as EVEScape. Limitations include the need for deeper analysis of feature contributions and further refinement of pre-training techniques.

Recent studies have proposed a range of evolution-informed and structure-aware mutation effect predictors. PETRA introduces trajectory-trained transformers for next-mutation prediction, while platforms such as previr.app integrate evolutionary fitness and antigenic forecasting in real-world surveillance settings^15,18^. Evolutionary likelihood models including DeepSequence and EVE leverage multiple sequence alignments and have demonstrated strong performance, particularly in small-data regimes^27^. Methods such as GEMME and Tranception combine evolutionary priors with machine learning, while structure-aware and inverse-folding models (e.g., ProtREM, SaESM2) incorporate three-dimensional constraints to assess mutation effects^28^.

Large-scale benchmarking efforts, including VenusMutHub, highlight that no single model dominates across all data regimes: evolutionary models excel when sequence diversity is limited, language models perform well in zero-shot settings, and structure-aware approaches improve functional interpretation^29^. In contrast to these methods, the proposed R-DELF framework integrates sequence, structure, phylogenetic context, and temporal evolution within a unified pipeline, enabling forward-looking mutation forecasting rather than static fitness estimation.

While direct benchmarking against all these methods is computationally prohibitive, our evaluation focuses on time-forward prediction and cross-module ablation to demonstrate complementary strengths rather than claiming universal superiority.

## Research gap

Ongoing evolution of viruses poses a threat to global health because recent ones tend to have a greater transmissibility and immune evasion, which reduces vaccine effectiveness. Current AI-based solutions that consider machine learning and deep learning are capable of detecting mutation patterns and providing support in vaccine adaptation, but they pose serious shortcomings. Moreover, most studies do not employ time-forward evaluation or strict chronological splitting, leading to high risks of data leakage where near-identical sequences appear in both training and testing. This compromises the reliability of reported performance. Poor sequencing of low-resource areas further eliminates diversity of datasets, which makes them weak in application globally^21^. Another major gap is the lack of benchmarking against strong, domain-relevant baselines such as EVEScape, DeepSequence, EVE, GEMME, or structure-aware models, which limits the scientific validity of many proposed approaches^27^. Existing works also rarely include transformer embeddings, or phylogenetic modelling, leaving the true source of performance gains unclear. Additionally, uncertainty quantification—critical for biological decision-making—is largely absent in current literature^33^. Furthermore, the current models are limited to the most common and well-studied viral family such as influenza and coronaviruses and are unable to extrapolate novel or zoonotic pathogens because of a dearth of prior data. The lack of uniform regulatory guidelines also increases such risks^25^. To fill these gaps, it is necessary to employ interdisciplinary work in the field of virology, bioinformatics, and data science. Further developments should aim at the improvement of data diversity, the inclusion of real-time surveillance of genomics, and explainable and ethically regulated AI systems. These advancements will make it possible to take the initiative in predicting viral evolution and vaccine preparedness globally with high reliability.

## Methodology

The suggested R-DELF (Refined Deep Evolutionary Learning Framework) integrates multiple biological information sources to predict viral evolutionary trajectories and assess vaccine suitability. The primary supervised task is time-forward per-residue mutation-risk prediction, where binary labels are assigned based on whether a residue mutates in future sequences unseen during training; variant-level risk and vaccine suitability are downstream, derived outcomes rather than direct prediction targets. The model is trained on SARS-CoV-2 genomic data capturing mutation patterns, temporal lineage dynamics, and evolutionary relationships. To incorporate structural constraints without requiring high-confidence 3D protein structures, R-DELF uses the Protein Secondary Structure 2022 dataset and ESM-2–derived inter-residue attention signals to generate structure-aware pseudo-contact priors. These priors’ approximate residue-level proximity and folding tendencies using sequence-derived information only. The ESM-2 Transformer produces contextual embeddings that capture long-range biochemical dependencies and co-evolutionary signals, which are further refined by the evolutionary learning and graph modules. R-DELF separates retrospective mutation classification from forward mutation risk ranking and vaccine suitability assessment, reporting accuracy only retrospectively while evaluating forward prediction using time-aware ranking and risk prioritization.

**Fig. 1:**
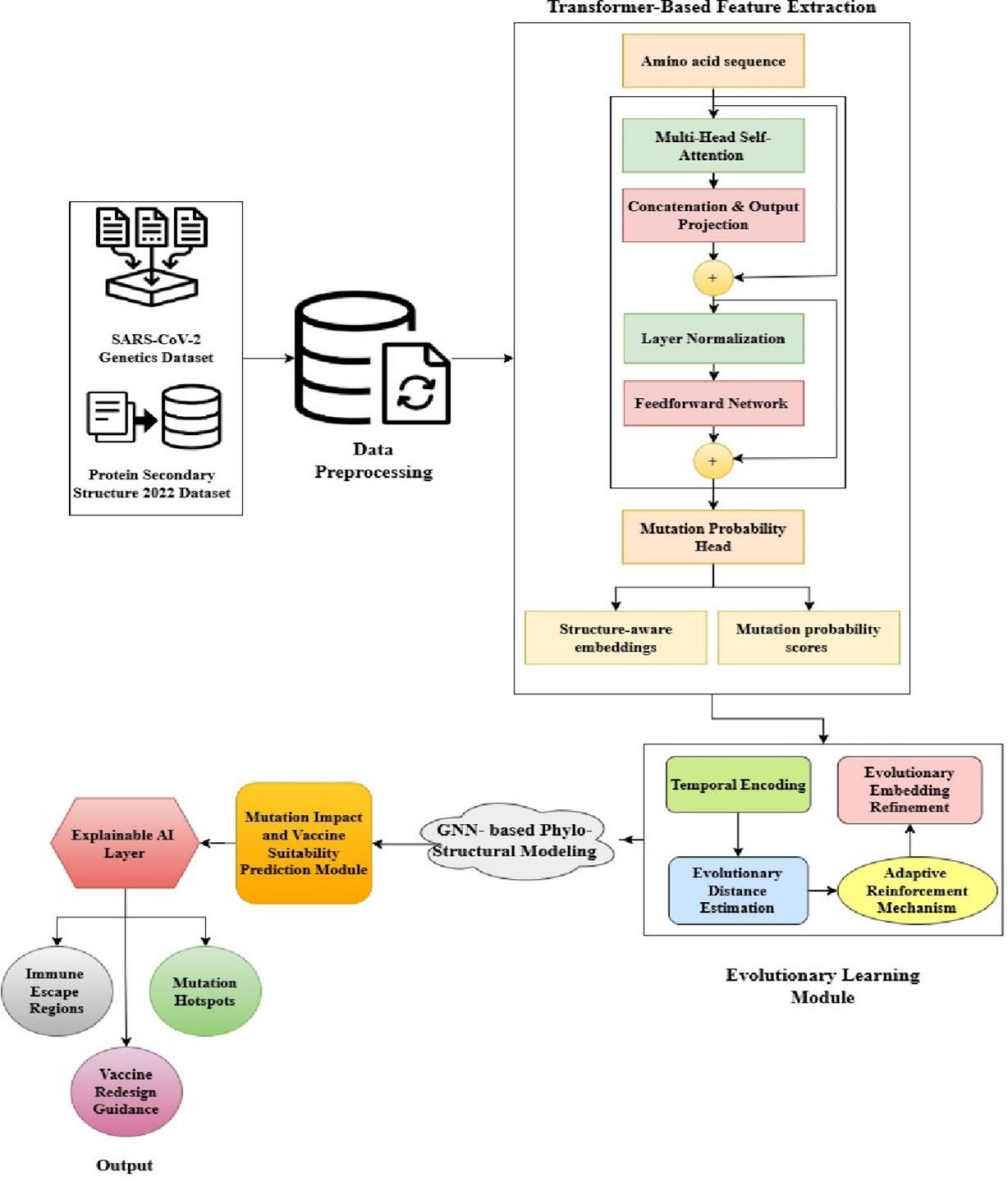
Overall Architecture of the Proposed R-DELF Framework.

In Fig. 1 the architecture integrates multi-source viral and structural datasets through a sequential pipeline. Cleaned and temporally normalized sequences are processed by the ESM-2 Transformer, generating structure-aware embeddings and mutation probabilities. These embeddings are refined through an Evolutionary Learning Module that incorporates temporal and adaptive reinforcement dynamics. A Dual-Attention GNN fuses phylogenetic and structural information to derive Structural Evolutionary Risk Score (SERS). The Mutation Impact and Vaccine Suitability Module Compute Functional Impact Score (FIS′), stability and binding alterations, and Pandemic Risk Rank (PRR). Finally, the XAI Layer consolidates attention, Shapley Additive Explanation (SHAP), and graph explanations into a Cross-Level Attribution Map (CLAM), providing transparent, biologically meaningful insights for vaccine design and pandemic preparedness.

Such embeddings are jointly trained with inferred probabilities of the secondary-structure, and are inputted into two GNNs- one phylogenetic graph of the lineage evolution model and one structural graph of the interaction of individual residues. An Evolutionary Learning Optimizer is a population based fine-tuner which enhances biological plausibility and predictive stability of the model. The last XAI module displays essential residues and clades involved in mutation threat making it easier to interpret to provide guidance on vaccines. The framework eventually converts the viral sequence information into an explicable, multi-layered intelligence model that is able to predict the presence of high-risk mutations and determine the location of immune escape regions that are both more biologically relevant and that have greater real-world applicability.

### Data collection

The data collection procedure implied the acquisition of genomic and proteomic sequences of SARS-CoV-2 and metadata of the Kaggle SARS-CoV-2 Genetics dataset and structural annotations of the Protein Secondary Structure 2022 dataset. These auxiliary datasets give sequence, temporal and structural data that are key in understanding viral evolution, predicting mutations, and identifying vaccine targets using structure in the R-DELF framework.

The data that is used as the main source of information of the viral genomics and proteomics is the SARS-CoV-2 Genetics data, which is retrieved through Kaggle^30^. It includes entire viral genome sequences, subunits of proteins and related metadata including date of collection, location of origin, phylogenetic lineage, and clade.

**Table 1:** SARS-CoV-2 genetics sample Data.

GenBank Accession ID	Genome Region (bp)	Gene/Protein	Protein Sequence Excerpt	Collection Date/Source Link
LC528233	21 571…25 392	Spike (S)	MFVFLVLLPLVSSQCVNLTTRTQLPPAYTNSFTRGVYYPDKVFRSSVLHSTQDLFLPFFSNVTWFHAIHVSGTNGT…	29 FEB 2020 NCBI link
MT370909	21513.25334	Envelope (E)	MFVFLVLLPLVSSQCVNLTTRTQLPPAYTNSFTRGVYYPDKVFRSSVLHSTQDLFLPFFSNVTWFHAIHVSGTNGTKRFDNPVLPFNDGVYFASTEKSNI…	23 APR NCBI link
MT292582	21 509…25 330	Spike (S)	MFVFLVLLPLVSSQCVNLTTRTQLPAYTNSFTRGVYYPDKVFRSSVLHSTQDLFLPFFSNVTWFHAIHVSGTNGT…	06 APR 2020 NCBI link

Table 1. shows five typical SARS-CoV-2 fragments of the genome on GenBank entries dated between February and April of 2020. It contains the sections of the genome, the corresponding proteins (Spike, Envelope, Membrane, etc.), the partial sequences of amino-acids, and links to the sources of NCBI. The following entries can illustrate the structure of the dataset, which is a mixture of sequence and metadata that is needed to track temporal mutations and model phylogenetic relationships. The information so curated allows the R-DELF framework to interpret patterns of mutations, evolution, and protein-level changes that would be highly important in forecasting the genetic adaptations that are relevant to vaccines.

The secondary-structure states of the proteins, i.e., Helix (H), Sheet (E), and Coil (C), are also annotated with the amino-acid sequences in the Protein Secondary Structure 2022 dataset, which is also referred to as Kaggle^31^. These structure annotations assist the R-DELF framework in reflecting the spatial dependencies that affect the stability of folding, antigenicity, and mutation tolerance. The uses within the framework include:

- Training a second-structure prediction auxiliary.
- Production of structure-sensitive residue-based features of SARS-CoV-2 proteins.
- Inputting node features on the structural graph GNN block.

**Table 2:** Sample Dataset — Protein secondary structure 2022.

PDB_ID	Chain_Code	Sequence (seq)	Secondary Structure (sst3)	Resolution (Å)
2EQ7	C	LAMPAAERLMQEKGVSPAEVQGTGLGGRILKEDVMRHLEE	CCCHHHHHHHHHCCCCCCCCCCCCCCCCCCHHHHCCCCCC	1.8
3A1G	B	GGSMERIKELRNLMSQSRTREILTKTTVDHMAIIKKYTSG	CHHHHHHHHHHHHCCCHHHHHHHHHCECCHHHHHHHCCCC	1.7
5D8V	A	AAPANAVTADDPTAIALKYNQDATKSERVAAARPGLPPEEQHCANCQFMQANVGEGDWKGCQLFPGKLINVNGWCASWTLKAG	CCCCCECCCCCHHHHHHCCECCHHHCCHHHHCCCCCCHHHCCHHHECCEEEEEEECCEEEECCCCCCEEECCCECCCCCECCC	0.48

Table 2 presents representative entries from the *Protein Secondary Structure 2022* dataset, showing protein identifiers (PDB_ID), chain codes, amino acid sequences, and their corresponding secondary structure classifications (Helix, Sheet, Coil) with X-ray crystallography resolution values. These data highlight the structural diversity of protein sequences, providing crucial information for residue-level modeling and enabling the proposed framework to learn structure-aware representations that enhance protein function prediction and SARS-CoV-2 mutation impact analysis.

### Data preprocessing

Prior to model integration into the R-DELF framework it is guaranteed that the data quality, uniformity and multi-modal compatibility are maintained through the data pre-processing pipeline. It combines the genomic, proteomic and structural data using a series of systematic cleaning, normalization, alignment and encoding. The datasets are used in this pipeline to prepare them to be used in transformer-based representation learning and GNN-based relational modeling.

#### Sequence cleaning and validation

Initially, all raw SARS-CoV-2 genome and protein sequences from the SARS-CoV-2 Genetics Dataset are validated for completeness and accuracy. Sequences containing ambiguous amino acid symbols (such as N, X, or gaps “–”) are filtered out. Furthermore, sequences that are too short or incomplete (less than 90% of the reference genome length) are discarded to maintain high data quality. Formally, a sequence $\:{S}^{\left(k\right)}$ is retained only if it satisfies, and derived in Eqs. (1),

1 $$\:\mathrm{retain}\left({S}^{\left(k\right)}\right){\hspace{0.25em}\hspace{0.05em}}\Leftrightarrow{\hspace{0.25em}\hspace{0.05em}}\frac{{n}_{\mathrm{amb}}\left({S}^{\left(k\right)}\right)}{{L}^{\left(k\right)}}\le\:{\tau}_{1}\mathrm{\:and\:}{L}^{\left(k\right)}\ge\:{\tau}_{2}\cdot\:{L}_{\mathrm{ref}}$$

where, $\:{n}_{\mathrm{amb}}\left({S}^{\left(k\right)}\right)$ is the number of ambiguous residues, $\:{L}^{\left(k\right)}$ is the sequence length, $\:{L}_{\mathrm{ref}}$ is the reference genome length, $\:{\tau\:}_{1}=0.01$, $\:{\tau\:}_{2}=0.9$. Duplicate sequences are removed using metadata comparison (date, location, and clade). Only human-host samples are retained to ensure biological consistency.

#### Temporal and structural feature normalization

The data combines both the temporal metadata (dates of collection) with structural priors (Protein Secondary Structure 2022) that are secondary. To record the evolutionary timelines (2) is used to normalize collection dates.

2 $$\:{t}^{\left(k\right)}=\frac{\mathrm{days}({d}^{\left(k\right)}-{d}_{\mathrm{m}\mathrm{i}\mathrm{n}})}{\mathrm{days}({d}_{\mathrm{m}\mathrm{a}\mathrm{x}}-{d}_{\mathrm{m}\mathrm{i}\mathrm{n}})}$$

where, $\:{d}_{\mathrm{m}\mathrm{i}\mathrm{n}}$ and $\:{d}_{\mathrm{m}\mathrm{a}\mathrm{x}}$ represent the most recent and the oldest times of sampling in the set. This scaling of the time progression between 0 and 1 normalizes this model and allows the identification of the patterns of mutations emergence as a function of time. Equation (3) estimates the probability of the secondary-structure of each residue $\:i$,

3 $$\:{s}_{i}=[{p}_{H},{p}_{E},{p}_{C}]$$

Representing likelihoods of Helix, Sheet and Coil. These are fed together with the Transformer embeddings $\:{e}_{i}$ as built in (4),

4 $$\:{\stackrel{\sim}{e}}_{i}=[{e}_{i}{\hspace{0.17em}}\parallel\:{\hspace{0.17em}}{s}_{i}]$$

The impact of mutation prediction is further improved by this embedding fusion which connects the context of the sequence with folding stability.

#### Training and evaluation

**Table 3:** Training and evaluation Dataset.

Sequences	Collection Date	Release Criteria	Count
Total SARS-CoV-2 GenBank Sequences	Jan 2020 – Dec 2022	All available	~ 10,000 + sequences*
Training Sequences (Pre-cutoff)	Jan 2020 – Jun 2021	Released before cutoff	~ 7,500 + sequences*
Validation Sequences (Mid period)	Jul 2021 – Dec 2021	Released within period	~ 1,500 + sequences*
Test Sequences (Future eval)	Jan 2022 – Dec 2022	Released after training period	~ 2,000 + sequences*

Table 3 shows the dataset statistics aggregated from the SARS-CoV-2 Genetics GenBank and Protein Secondary Structure 2022 Kaggle datasets. The lists include genomic and protein sequences with associated metadata spanning Jan 2020–Dec 2022 for time-forward prediction. Exact counts approximate availability from the original data sources. *Approximate counts based on dataset naming & descriptions. †Protein secondary structure dataset sizes historically reported around ~ 9,000 entries.

The curated dataset is partitioned using a chronological train–validation–test strategy to reflect the temporal nature of viral evolution. Sequences collected up to mid-2021 are used for model training, while sequences from late 2021 are reserved for validation. All sequences collected during 2022 are held out exclusively for testing. To avoid data leakage, training and test splits were separated by collection date, and phylogenetically related descendant sequences were restricted to the same temporal partition. Structural priors and secondary-structure annotations were independent of mutation labels and did not encode future evolutionary information.

### Feature extraction via transformer encoder

The first stage of the proposed R-DELF framework is the feature extraction process that is aimed at converting linear amino acid sequences into rich and context-aware embeddings, which would retain both biochemical- and evolutionary-level information. It uses the state-of-the-art protein language model called the ESM-2 Transformer which is trained on millions of protein sequences and is able to learn deep representations of residue interactions.

The proposed R-DELF framework employs the ESM-2 (650 M parameter) protein language model as the Transformer backbone for sequence representation. Protein sequences are truncated or padded to a maximum length of 1,024 amino acid residues, covering the full length of SARS-CoV-2 structural proteins while maintaining computational efficiency. To preserve pretrained biochemical and evolutionary knowledge, ESM-2 weights are frozen during initial training, and only downstream layers—including the mutation prediction head, evolutionary learning module, and graph neural network—are optimized. In a secondary fine-tuning phase, the top 4 Transformer layers are unfrozen to enable limited task-specific adaptation while avoiding overfitting to viral lineage redundancy. Optimization is performed using the Adam optimizer with a base learning rate of 1 × 10⁻⁴ for downstream modules and 1 × 10⁻⁵ for unfrozen ESM-2 layers. A cosine decay learning-rate scheduler is applied, and gradient clipping is used to stabilize training. This hybrid freezing strategy balances biological generalization with mutation-specific sensitivity.

**Fig. 2:**
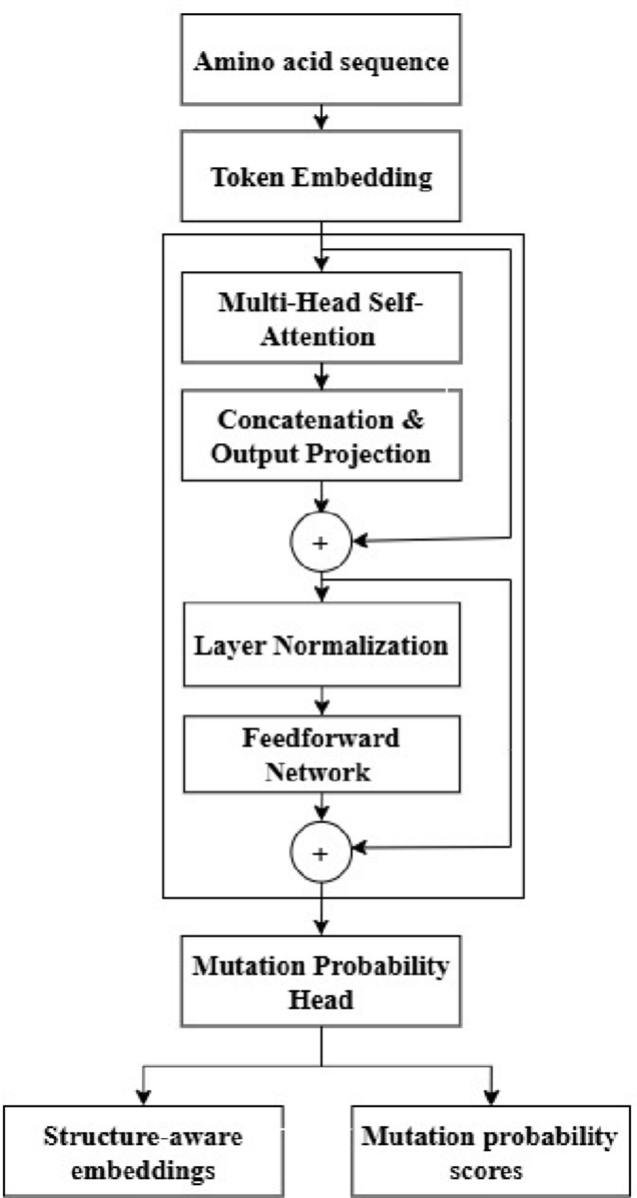
Architecture Diagram of ESM-2 Transformer.

Figure 2 demonstrates that the ESM-2 Transformer architecture can be used to transform amino acid sequences into high-dimensional embeddings, which are conditioned by biochemical and evolution contexts. It is a short and long-range residue dependency model based on multi-head self-attention. These embeddings are further integrated with secondary-structure priors in order to bring in the concept of space awareness.

#### Input representation

In the suggested R-DELF model, every viral or protein sequence is handled as a sequence of amino acid residues in an ordered collection based on the aligned dataset. Formally, a sequence of proteins is expressed in the following (5),

5 $$\:{S}^{\left(k\right)}=({a}_{1}^{\left(k\right)},{a}_{2}^{\left(k\right)},..,{a}_{L}^{\left(k\right)})$$

where $\:{a}_{i}^{\left(k\right)}$ represents the residue of sequence k in position i, and L-represents the entire sequence length. The tokenizer ESM-2 is used to convert each scarce amino acid token a i (k) into a high dimensional embedding as in Eqs. (6),

6 $$\:{h}_{i}^{\left(0\right)}=E\left({a}_{i}^{\left(k\right)}\right)+{P}_{i}$$

where, $\:E(\cdot\:)$ refers to the learning embedding operation taking the amino acids and producing d-dimensional dense vectors, which encode the biochemical and evolutionary attributes of the amino acids. $\:{P}_{i}$ represents the positional encoding vector, which preserves the sequential order of residues within the protein chain. The combined embedding $\:{h}_{i}^{\left(0\right)}$thus integrates semantic, spatial, and contextual information, forming the foundational input for the Transformer encoder in the feature extraction stage.

#### Multi-Head Self-Attention mechanism

The core mechanism of the Transformer architecture is the Multi-Head Self-Attention (MHSA), which allows the model to learn dependencies between amino acids regardless of their distance in the sequence. This is crucial in proteins, where residues distant in sequence may interact structurally. For each attention head $\:h\in\:\{\mathrm{1,2},...,H\}$, the model computes query, key, and value vectors. The attention score between residues $\:i$and $\:j$determines how much attention residue $\:i$should pay to residue $\:j$when forming its contextual representation derived in Eqs. (7),

7 $$\:{\alpha\:}_{ij}^{\left(h\right)}=\mathrm{softmax}\left(\frac{\left({Q}_{i}^{\left(h\right)}\right)({K}_{j}^{\left(h\right)}{)}^{T}}{\sqrt{{d}_{h}}}\right)$$

where, the softmax operation normalizes the attention scores so that they sum to one, providing a probability distribution over residues. The scaled dot-product ensures numerical stability and balances gradient magnitudes during training. The contextual output for residue $\:i$from head $\:h$ is computed using Eqs. (8),

8 $$\:{z}_{i}^{\left(h\right)}=\sum\:_{j=1}^{L}{\alpha\:}_{ij}^{\left(h\right)}{V}_{j}^{\left(h\right)}$$

where, $\:{z}_{i}^{\left(h\right)}$ represents the weighted combination of all residues’ value vectors according to their attention importance. The results of the sum of all the $\:H$ attention heads are then linearly transformed and the result is the final embedding of residue $\:i$ generated by the Eqs. (9),

9 $$\:{e}_{i}={W}_{O}[{z}_{i}^{\left(1\right)}\parallel\:{z}_{i}^{\left(2\right)}\parallel\:...\parallel\:{z}_{i}^{\left(H\right)}]$$

where, $\:{W}_{O}\in\:{\mathbb{R}}^{d\times\:(H\cdot\:{d}_{h})}$ is the output projection matrix, and “$\:\parallel\:$”represents head concatenation. This is the mechanism by which ESM-2 can simultaneously process a variety of biochemical and evolutionary interactions, effectively describing multi-residue interactions within the protein sequence.

#### Transformer encoder layer composition

The ESM-2 Transformer encoder type has two critical sublayers per layer, the MMHA mechanism and the Position-wise FFN, and each of them is enclosed by residual connections and layer normalization to maintain gradient flow stability and enhanced learning performance. The computation of the layer can be written as in (10),

10 $$\:{h}_{i}^{\left(l\right)}=\mathrm{LayerNorm}({h}_{i}^{\left(l-1\right)}+\mathrm{FFN}\mathrm{(}\mathrm{LayerNorm}\mathrm{(}({h}_{i}^{\left(l-1\right)}+\mathrm{MHA}\left({\mathrm{h}}^{\left(l-1\right)}\right)\left)\right))$$

where, $\:{h}_{i}^{(l-1)}$ denotes the embedding that the last layer left behind, $\:\mathrm{MHA}(\cdot\:)$ is contextual attention among the residues, and $\:\mathrm{FFN}(\cdot\:)$ is non-linear transformation to richer the input, and $\:LayerNorm(\cdot\:)$ normalizes the input to make the training process stable. The FWN optimizes the attention outputs as current (11),

11 $$\:\mathrm{FFN}\left(x\right)={W}_{2}\cdot\:\mathrm{ReLU}({W}_{1}x+{b}_{1})+{b}_{2},$$

where $\:{W}_{1},{W}_{2}$ are learnable weight matrices, $\:{b}_{1},\:{b}_{2}$ are bias terms, and ReLU is used to add non-linearity, to learn the complicated residue relationships. Following a sequence of $\:{L}_{T}$ stacked Transformer layers, the resulting contextual coding of residue $\:i$ in sequence $\:k$ is defined by the following equation, referred to as Eqs. (12),

12 $$\:{e}_{i}^{\left(k\right)}={h}_{i}^{\left({L}_{T}\right)}\in\:{\mathbb{R}}^{d}$$

where $\:{L}_{T}$ is the number of the layers of the encoder and dis the embedding dimension. These embeddings represent both local interaction of the residues and global sequence interactions that are important to mutation prediction.

#### Structural context enhancement

In order to combine spatial awareness, every contextual embedding $\:{e}_{i}^{\left(k\right)}$ is augmented by the secondary-structure priors obtained by means of Protein Secondary Structure 2022 dataset. The probabilistic structure of the individual amino acid is a probability vector as shown in the Eqs. (13),

13 $$\:{s}_{i}^{\left(k\right)}=\left[{p}_{H},{p}_{E},{p}_{C}\right]$$

where, $\:{p}_{H}$, $\:{p}_{E}$, and $\:{p}_{C}\:$are the probability of the residue to be a part of a Helix, Sheet, and Coil respectively. This vector is concatenated with sequence embedding, which is obtained in (14) to obtain the enhanced structure-aware embedding.

14 $$\:{\stackrel{\sim}{e}}_{i}^{\left(k\right)}=[{e}_{i}^{\left(k\right)}{\hspace{0.25em}\hspace{0.05em}}\parallel\:{\hspace{0.25em}\hspace{0.05em}}{s}_{i}^{\left(k\right)}]$$

The combination of both secondary-structure priors and sequence semantics in this fusion permits the model to make more sensible predictions of both residue interactions and stability factors of functional interest.

#### Contextual mutation probability Estimation

After contextual and structural embeddings have been made, the R-DELF architecture uses a self-predictive mutation head to estimate the likelihood of a mutation in each residue using the following (15),

15 $$\:{p}_{i}=\sigma\:\left({W}_{m}{E}_{i}+{b}_{m}\right)$$

where, $\:{W}_{m}$ and $\:{b}_{p}$ are learnable parameters, $\:\sigma\:(\cdot\:)$ is the sigmoid activation function.

##### Algorithm 1

Feature Extraction via Transformer Encoder (ESM-2-based).


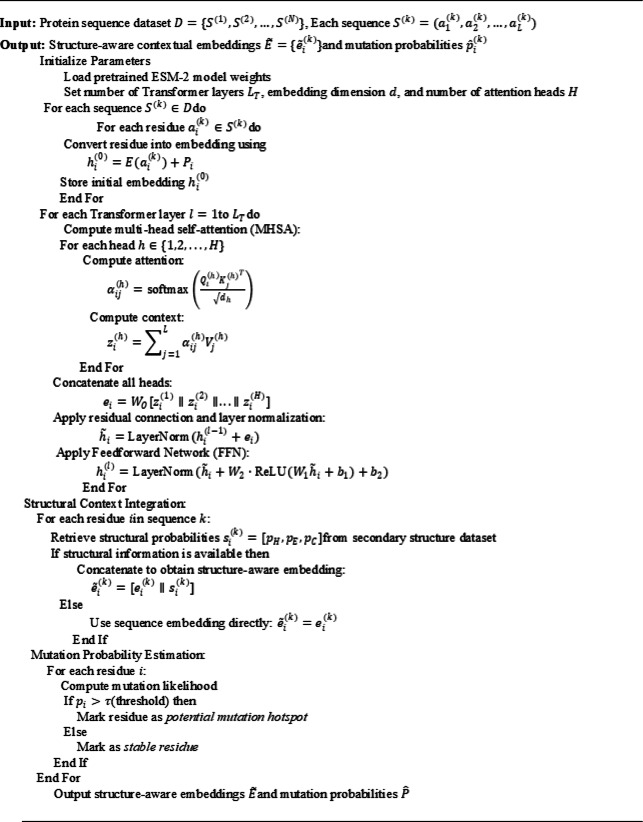


Algorithm 1 is a sequence of transformations through which the raw amino acid sequences are turned into high dimensional embeddings with the ESM-2 Transformer. The model is able to capture intricate dependencies among the residues via multi-head attention, feedforward refinement and secondary-structure fusion. The mutation prediction head, in turn, measures the probability of mutational changes at every residue, becoming the input of Evolutionary Learning and GNN-based phylogenetic analysis in the further steps.

### Evolutionary learning module

The proposed R-DELF framework Evolutionary Learning Module describes the dynamics of evolutionary mutations over time and viral lineages through the modeling of temporal dependencies, evolutionary distances, and biologically beneficial mutations. It refines the Transformer-derived structure-aware embeddings $\:{\stackrel{\sim}{e}}_{i}^{\left(k\right)}$into evolution-aware representations, and thus allowing the model to acquire knowledge of the process of creating mutations, which spread and survive due to the applied selective pressure.

#### Temporal encoding

To explicitly incorporate chronological progression, we augment the ESM-2 embedding with a learnable temporal projection in (16),

16 $$\:{E}_{i}^{{\prime\:}}\left(t\right)={E}_{i}+g{T}_{i}$$

where, $\:{E}_{t}$ is the ESM-2 residue embedding, $\:{T}_{t}$ is the normalized collection time, $\:PE\left({T}_{t}\right)$ is the learnable coefficient that controls the contribution of temporal evolution. Such an encoding enables the framework to capture mutation dynamics over time so that recent and old strains are put in place in the embedding space.

#### Evolutionary distance Estimation

In the analysis of phylogenetic divergence between viral variants, pairwise evolutionary distances between viand vajra strains were calculated, based on cosine dissimilarity calculated in (17),

17 $$\:\begin{array}{cccc}&\:D({v}_{i},{v}_{j})=1-\frac{{E}_{u}^{{\prime\:}}\cdot\:{E}_{v}^{{\prime\:}}}{\parallel\:{E}_{u}^{{\prime\:}}\parallel\:\parallel\:{E}_{v}^{{\prime\:}}\parallel\:}&\:&\:\end{array}$$

where $\:{E}_{i}^{{\prime\:}}$and $\:{E}_{j}^{{\prime\:}}$ are temporal representations of two viral strains. A large value of $\:D({v}_{i},{v}_{j})$ is the embedding of the strain at time t which is the normalized collection time Tt is the normalized collection time, g is the learnable coefficient that controls the contribution of temporal evolution. Such an encoding enables the framework to capture mutation dynamics over time so that recent and old strains are put in place in the embedding space.

#### Adaptive mutation reinforcement

In order to highlight biologically significant mutations an adaptive reinforcement signal $\:{R}_{t}$ was added. This signal acts in favour of mutations that enhance the mutation rate, as well as that follow an optimal evolutionary path in (18),

18 $$\:\begin{array}{cccc}&\:{R}_{t}=\alpha\:\cdot\:{\Delta\:}{p}_{t}+\beta\:\cdot\:(1-D({v}_{t-1},{v}_{t}\left)\right)&\:&\:\text{}\end{array}$$

where $\:{\Delta\:}{p}_{t}={p}_{t}-{p}_{t-1}$ is the difference between mutation probability of successive time points, $\:D({v}_{t-1},{v}_{t})$ is the evolutionary distance between the consecutive viral variants, a and b are the balancing coefficients of mutation strength and lineage continuity. When $\:{R}_{t}$ exceeds a set adaptive threshold th, the mutation is an advantageous one, otherwise, it is a neutral mutation or a deleterious mutation. The mechanism of reinforcement allows the framework to emphasize selectively beneficial mutations - those that continue and outcompete with evolutionary time-scale - to enhance the predictive power of the model in emergence of variants.

##### Evolutionary learning optimizer (ELO)

The Evolutionary Learning Optimizer (ELO) refines the Transformer-derived embeddings by incorporating temporal progression, evolutionary divergence, and adaptive reinforcement signals that highlight biologically advantageous mutations. This subsection formalizes the mathematical operations of ELO.

**a. Temporal Evolution Embedding**.

For each viral strain sampled at time $\:t$, its base sequence embedding from the ESM-2 model is denoted as (19),

19 $$\:{E}_{t}={f}_{\mathrm{ESM2}}\left({x}_{t}\right)$$

To incorporate chronological evolution, a temporal encoding proportional to normalized sampling time $\:{\tau\:}_{t}$ is added using (20),

20 $$\:{E}_{t}^{\left(temp\right)}={E}_{t}+g\cdot\:{\tau\:}_{t}$$

where, $\:{\tau\:}_{t}\in\:\left[\mathrm{0,1}\right]$ is the normalized collection time, and $\:g$ is a learnable coefficient controlling temporal influence.

**b. Evolutionary Distance Between Successive Strains**.

The divergence between two consecutive strains is quantified using cosine dissimilarity in (21),

21 $$\:{D}_{t,t+1}=1-\mathrm{c}\mathrm{o}\mathrm{s}({E}_{t}^{\left(temp\right)},{\hspace{0.17em}}{E}_{t+1}^{\left(temp\right)})$$

A higher value indicates greater evolutionary drift.

**c. Adaptive Reinforcement Signal**.

For each residue or strain-level prediction, let $\:{p}_{t}$and $\:{p}_{t+1}$denote the predicted mutation probabilities at times $\:t$and $\:t+1$. ELO assigns a reinforcement value based on both mutation gain and lineage continuity in (22)

22 $$\:{R}_{t}=\alpha\:{\hspace{0.17em}}({p}_{t+1}-{p}_{t})-\beta\:{\hspace{0.17em}}{D}_{t,t+1}$$

where, $\:\alpha\:\:$controls the impact of mutation strength, $\:\beta\:$ penalizes abrupt evolutionary jumps. A mutation is considered adaptive when $\:{R}_{t}\ge\:\theta\:$, with $\:\theta\:$being an adaptive threshold learned during training.

**d. Evolution-Aware Embedding Update**.

The embedding at time $\:t+1$is refined by integrating the reinforcement signal in (23),

23 $$\:{\stackrel{\sim}{E}}_{t+1}={E}_{t+1}^{\left(temp\right)}+\lambda\:{R}_{t}$$

where, $\:\lambda\:$ regulates the reinforcement contribution. $\:{\stackrel{\sim}{E}}_{t+1}$ becomes the final evolution-aware representation used by downstream GNN layers.

#### Evolutionary learning output

A mutation embedding is a temporally contextualised mutation embedding that is the output of the Evolutionary Learning Module computed via $\:{R}_{t}\cdot\:{E}_{t}^{{\prime\:}}$. To characterize mutation dynamics across time, further improvement is performed on the Transformer-based embeddings used by adding the concepts of temporal progression, evolutionary distance, and adaptive reinforcement in the Evolutionary Learning Module.

To characterize mutation dynamics across time, further improvement is performed on the Transformer-based embeddings used by adding the concepts of temporal progression, evolutionary distance, and adaptive reinforcement in the Evolutionary Learning Module. Temporal encoding can put viral strains into chronological order; cosine distance is used to measure variability of the variants and reinforcement selects mutations that are biologically beneficial. This integration makes the fixed protein embeddings moving towards dynamic representations, both structural and adaptive evolution. The context-enhanced embeddings of the resultant capture the biological meaning and stability of mutations as optimized to be used as graph-based phylogenetic models in the next phase of the R-DELF-framework.

### Graph neural network-based phylogenetic modeling

The R-DELF framework integrates evolutionary intelligence with structure-aware priors through the GNN-based Phylo-Structural Modeling module. Instead of relying on experimentally determined 3D structures, R-DELF uses predicted pseudo-contact signals derived from ESM-2 attention patterns and secondary-structure consistency. These signals approximate fold-level constraints and allow the model to evaluate whether predicted mutations are likely to be structurally compatible. By combining evolutionary trajectories with these structure-aware pseudo-contacts, R-DELF captures mutation feasibility and functional impact more accurately, enabling more reliable identification of variants with potential immune escape or vaccine sensitivity.

#### Graph construction from evolutionary and structural signals

Assume that each variant $\:{v}_{i}\:$(of evolutionary learning) is embedded in $\:{E}_{i}^{{\prime\:}{\prime\:}}\in\:{\mathbb{R}}^{d}$ and a structural descriptor $\:{S}_{i}\in\:{\mathbb{R}}^{3}$. The phylo-structural adjacency matrix A is a combination of the evolutionary proximity and the structural similarity by means of the following Eq. (24),

24 $$\:\begin{array}{cccc}&\:{A}_{ij}=\begin{array}{c}\underbrace{{\mathrm{exp}\left(-{\gamma\:}_{d}D\left({v}_{i},{v}_{j}\right)\right)}}\\\:\mathrm{e}\mathrm{v}\mathrm{o}\mathrm{l}\mathrm{u}\mathrm{t}\mathrm{i}\mathrm{o}\mathrm{n}\text{}\mathrm{d}\mathrm{i}\mathrm{s}\mathrm{t}\mathrm{a}\mathrm{n}\mathrm{c}\mathrm{e}\end{array}\mathrm{+}\begin{array}{c}\underbrace{{{\eta\:}_{s}\mathrm{exp}\left(-{\gamma\:}_{s}\parallel\:{S}_{i}-{S}_{j}{\parallel\:}_{2}^{2}\right)}}\\\:\mathrm{s}\mathrm{t}\mathrm{r}\mathrm{u}\mathrm{c}\mathrm{t}\mathrm{u}\mathrm{r}\mathrm{e}\text{}\mathrm{s}\mathrm{i}\mathrm{m}\mathrm{i}\mathrm{l}\mathrm{a}\mathrm{r}\mathrm{i}\mathrm{t}\mathrm{y}\end{array}{\hspace{0.25em}\hspace{0.05em}}&\:&\:\end{array}$$

where $\:D({v}_{i},{v}_{j})$ is the evolutionary distance from Eq. (24), and $\:{\gamma\:}_{d},{\gamma\:}_{s},{\eta\:}_{s}$ are scaling constants. A weighted graph $\:G=(V,E,A)$ representing both sequence and conformation coupling is obtained by this combination adjacency.

#### Multi-Modal node initialization

The first feature vector of each node combines evolutionary, mutation, and structural representations with the help of the following Eqs. (25),

25 $$\:\begin{array}{cccc}\:&\:{x}_{i}^{\left(0\right)}=\left[{E}_{i}^{{\prime\:}{\prime\:}}\parallel\:{s}_{i}\parallel\:{p}_{i}\right]&\:&\:\end{array}$$

where, $\:{E}_{i}^{{\prime\:}{\prime\:}}\:$is the evolution-conscious embedding of the evolutionary learning module, is the probability vector of the secondary-structure, is the probability vector $\:[{p}_{H},{p}_{E},{p}_{C}]$, $\:{p}_{i}$ is the mutation likelihood of the Transformer head. The combination of these elements enables the model to consider structural stability and evolutionary adaptiveness as mutually enhancement objectives in learning.

#### Dual-Attention message passing

Dual-Attention Graph Neural Network (DAGNN) in the R-DELF framework performs the tasks of combining two independent relationships between viral variants, including Phylogenetic dependencies, and Structural dependencies. This two-fold modelling allows the evolutionary and structural adaptation to be learnt together, which offers a more comprehensive picture of the viral mutation spread. Phylogenetic attention identifies how much influence neighboring variants (in time or lineage) exert on node $\:{v}_{i}$. For node $\:{v}_{i}$, its neighbors $\:j\in\:\mathcal{N}\left(i\right)$ are defined by the evolutionary adjacency.

To formally define the dual-attention mechanism, we first compute two independent attention scores: one capturing phylogenetic influence and another capturing structural proximity. For any node $\:{v}_{i}$and its neighbor $\:{v}_{j}$, the phylogenetic attention weight is given by (26),

26 $$\:{\alpha\:}_{ij}^{phylo}={\mathrm{softmax}}_{j}\left({a}_{p}^{T}\left[{W}_{p}{h}_{i}{\hspace{0.17em}}\parallel\:{\hspace{0.17em}}{W}_{p}{h}_{j}\right]\right)$$

where, $\:{h}_{i},\:and\:{h}_{j}$ are the input feature vectors of nodes $\:i$ and $\:j$ from the previous GNN layer. The structural attention weight is computed as (27),

27 $$\:{\alpha\:}_{ij}^{struct}={\mathrm{softmax}}_{j}\left({a}_{s}^{T}\left[{W}_{s}{h}_{i}{\hspace{0.17em}}\parallel\:{\hspace{0.17em}}{W}_{s}{h}_{j}\right]\right)$$

where, $\:{W}_{s}$ is the learnable projection matrix for structural embeddings, $\:{a}_{s}$ is learnable attention vector for structural relationships, and $\:{\alpha\:}_{ij}^{struct}$ is the structural attention weight reflecting how similar the structural conformations of variants $\:i$ and $\:j$ are. The combined attention-guided update for node $\:{v}_{i}$ at layer $\:l$ is expressed as (28),

28 $$\:{h}_{i}^{{\prime\:}}=\sigma\:\left(\gamma\:\sum\:_{j\in\:N\left(i\right)}{\alpha\:}_{ij}^{phylo}{\hspace{0.17em}}{W}_{p}{h}_{j}+(1-\gamma\:)\sum\:_{j\in\:N\left(i\right)}{\alpha\:}_{ij}^{struct}{\hspace{0.17em}}{W}_{s}{h}_{j}\right)$$

where, $\:0\le\:\gamma\:\le\:1$ controls the fusion between the two attention channels. Similarly, structural attention learns how compatible or correlated two variants are based on their predicted structural proximity, derived in (29),

29 $$\:{\alpha\:}_{ij}^{S}=\frac{\mathrm{e}\mathrm{x}\mathrm{p}\left(\mathrm{LeakyReLU}\right({a}_{S}^{T}[{W}_{S}{S}_{i}{\hspace{0.17em}}\parallel\:{\hspace{0.17em}}{W}_{S}{S}_{j}]\left)\right)}{\sum\:_{k\in\:\mathcal{N}\left(i\right)}\mathrm{e}\mathrm{x}\mathrm{p}\left(\mathrm{LeakyReLU}\right({a}_{S}^{T}[{W}_{S}{S}_{i}{\hspace{0.17em}}\parallel\:{\hspace{0.17em}}{W}_{S}{S}_{k}]\left)\right)}$$

where, $\:{W}_{S}\in\:{\mathbb{R}}^{{d}_{s}\times\:3}$ is the projection for structural embeddings, and $\:{a}_{S}\in\:{\mathbb{R}}^{2{d}_{s}}\:$is the learnable attention vector. This learns which variants have structural stability correlations. Even if two variants are far apart in time, if their proteins fold similarly, they share structural attention. Each node aggregates messages from neighbours using a weighted fusion of both attentions, derived in (30),

30 $$\:{h}_{i}^{\left(l\right)}=\sigma\:(\sum\:_{j\in\:\mathcal{N}\left(i\right)}({\lambda\:}_{P}{\alpha\:}_{ij}^{P}+{\lambda\:}_{S}{\alpha\:}_{ij}^{S}){\hspace{0.17em}}{W}_{h}^{\left(l\right)}{h}_{j}^{(l-1)})$$

where, $\:{\lambda\:}_{P},{\lambda\:}_{S}\:$ is the trainable balance coefficients, $\:{W}_{h}^{\left(l\right)}\in\:{\mathbb{R}}^{d\times\:d}$ is the layer-specific transformation matrix, and the $\:\sigma\:(\cdot\:)$ is a non-linear activation (ReLU or GELU).

#### Phylo-Structural lineage and risk inference

Each variant has its evolutionary-structural identity, which is expressed by the final embedding. With these embeddings, R-DELF makes predictions of the lineage transitions and the possibility of vaccine escape as in (31),

31 $$\:P({v}_{j}\mid\:{v}_{i})=\mathrm{softmax}\left({W}_{m}\right[{h}_{i}^{\left(L\right)}\parallel\:{h}_{j}^{\left(L\right)}\left]\right)$$

where, $\:{h}_{i}^{\left(L\right)}\:$ is the last embeddings and Structural Evolutionary Risk Score (SERS) is obtained by following the (32),

32 $$\:SER{S}_{i}=\sigma\:\left({W}_{r}\right[{h}_{i}^{\left(L\right)}\parallel\:{S}_{i}]+{b}_{r})$$

A high SERS value represents a divergent variant that is both structurally divergent and evolutionarily adaptable to signalling may represent immune evasion.

#### Composite loss function

The optimization of GNN is a combination of accuracy of mutation, phylogenetic stability, and structural coherence, which is obtained in (33),

33 $$\:{\mathcal{L}}_{GNN}={\mathcal{L}}_{mut}+{\lambda\:}_{1}{\mathcal{L}}_{phylo}+{\lambda\:}_{2}{\mathcal{L}}_{cons}$$

where, $\:{\mathcal{L}}_{mut}$ is the mutation classification loss, $\:{L}_{phylo}$ the evolutionary consistency loss and $\:{L}_{cons}$ temporal + structural smoothness penalty.

##### Mutation classification loss

This term ensures the GNN correctly predicts mutation-prone and mutation-stable residues using (34),

34 $$\:{\mathcal{L}}_{mut}=-\sum\:_{i}\left[{y}_{i}\mathrm{l}\mathrm{o}\mathrm{g}{p}_{i}+(1-{y}_{i})\mathrm{l}\mathrm{o}\mathrm{g}(1-{p}_{i})\right]$$

where, $\:{y}_{i}$ is the true mutation label, and $\:{p}_{i}$ is the predicted mutation probability from the Transformer-GNN stack.

##### Evolutionary consistency loss

This regulates agreement between predicted and empirical phylogenetic distances using (3),

35 $$\:{\mathcal{L}}_{phylo}=\sum\:_{(i,j)\in\:\varepsilon}{\left({D}_{ij}^{pred}-{D}_{ij}^{tree}\right)}^{2}$$

where, $\:{D}_{ij}^{tree}$ is the patristic distance from reference phylogenetic tree, and $\:{D}_{ij}^{pred}$= is predicted embedding distance (e.g., cosine dissimilarity). This prevents the GNN from learning biologically implausible variant relationships.

##### Structural smoothness loss

Ensures that structurally similar variants maintain compatible embeddings using (36),

36 $$\:{\mathcal{L}}_{struct}=\sum\:_{i,j}{A}_{ij}^{struct}{\parallel\:{h}_{i}^{\left(L\right)}-{h}_{j}^{\left(L\right)}\parallel\:}^{2}$$

where, $\:{A}_{ij}^{struct}$ is the structural adjacency derived from pseudo-contact maps or attention-derived distances. This stabilizes the learned representation around realistic protein-folding constraints.

##### Temporal continuity loss

Ensures smooth embedding transitions across chronological variant progression using (37)

37 $$\:{\mathcal{L}}_{temp}=\sum\:_{t}\parallel\:{H}_{t}-{H}_{t-1}{\parallel\:}^{2}$$

The phylo-structural GNN models mutation dynamics through dual-attention, ensuring temporal and structural consistency, and produces SERS scores identifying variants with high evolutionary adaptability, functional impact, and immune-escape potential for early-risk prediction.

###### Algorithm 2

Phylo-Structural GNN Process.


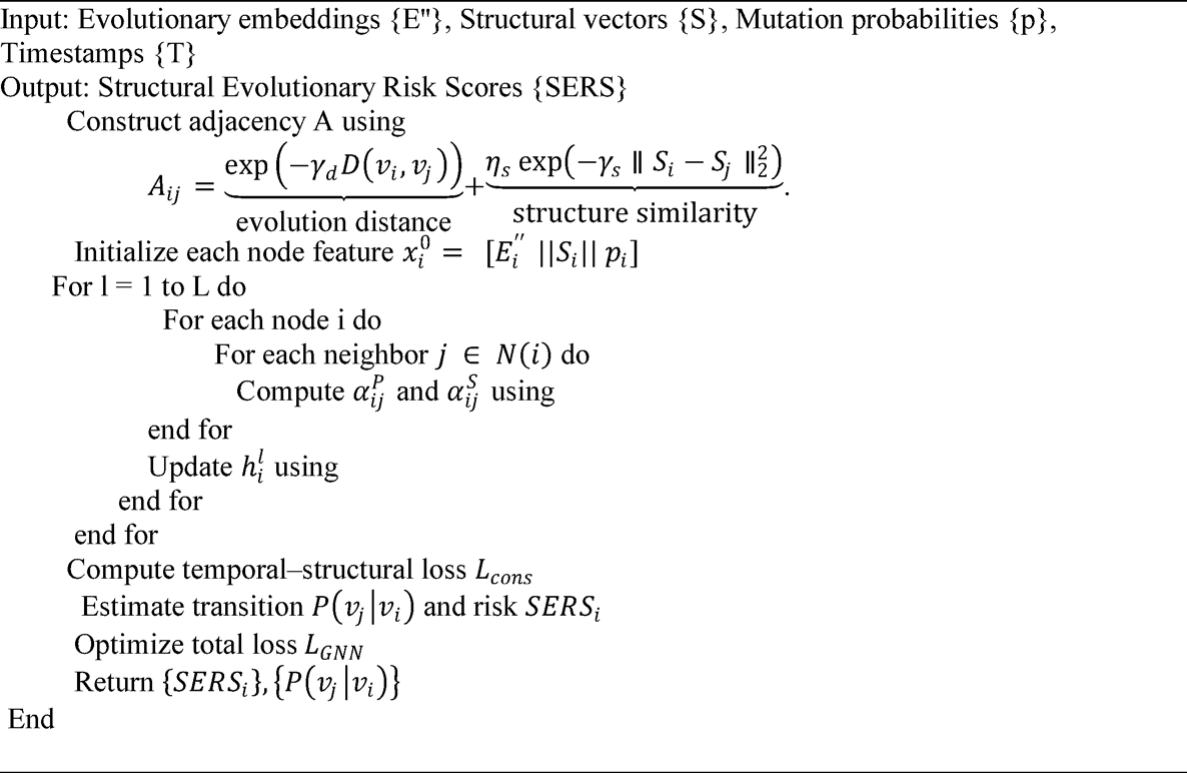


Algorithms 2 shows the evolutionary, structural and temporal data is combined in algorithm 2 to model the dynamics of the viral mutation evolution process and the effects of these mutations on the stability of proteins. It starts by assembling a phylo-structural adjacency graph, with each node an individual viral strain having joint embeddings of mutation, structure and time. Attention weights are calculated by a multi-layer GNN propagation to obtain both a phylogenetic and structural relationship. The model reduces a loss on time-structural, and approximates the transition probabilities of mutations and SERS to determine high-risk viral variants.

**Fig. 3:**
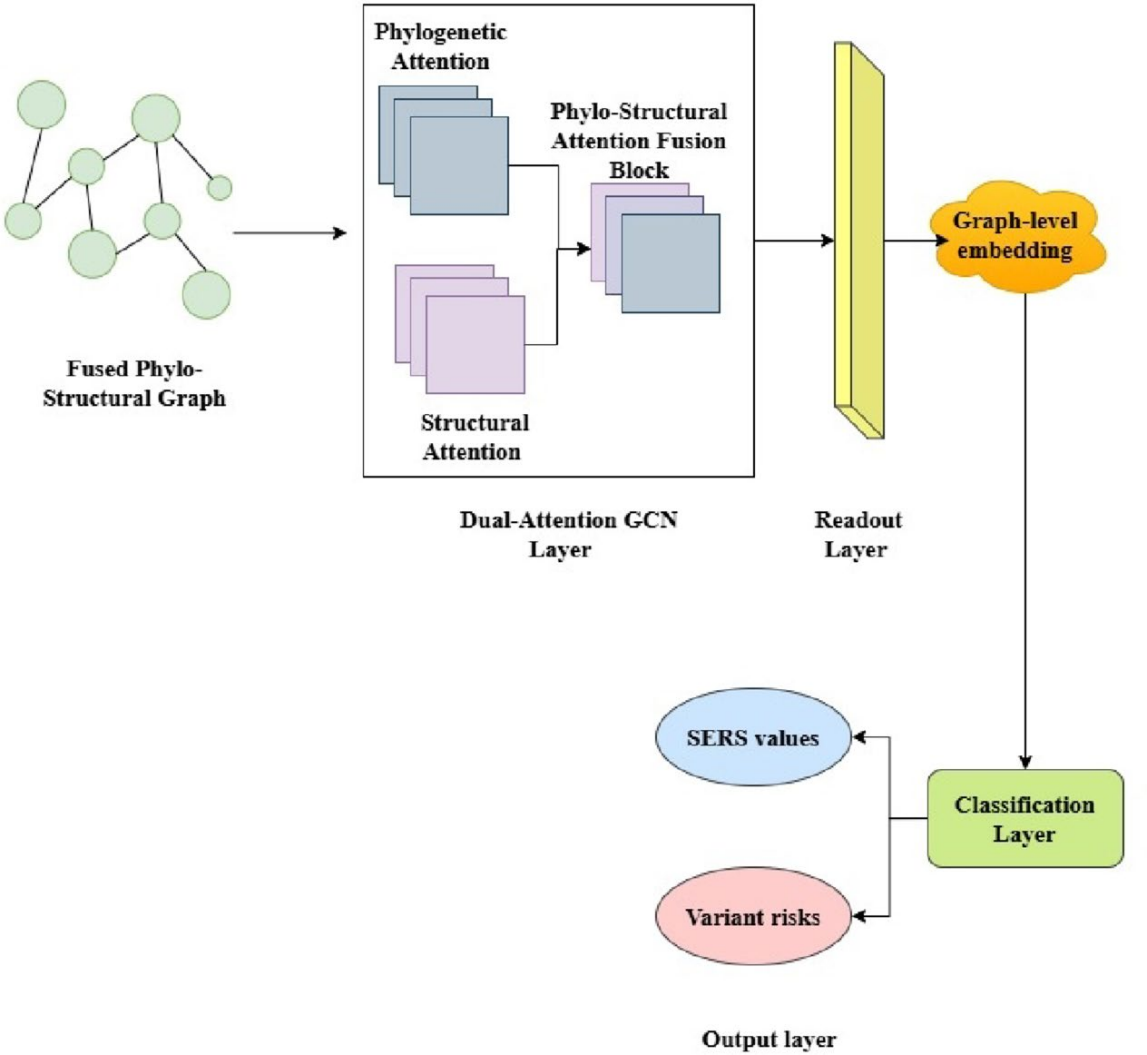
Overall Architecture of the Proposed R-DELF Framework.

Figure 3 has undertaken a step-by-step sequence of connecting multi-source viral and structural datasets into an integrated format. The ESM-2 Transformer then takes in cleaned and temporally normalized sequences to produce structure-aware embeddings and mutation probabilities. These embeddings are trained by a refinement process, an Evolutionary Learning Module, which integrates a time and adaptive reinforcement dynamic mechanism. A Dual-Attention GNN combines both phylogenetic and structural data to come up with SERS.

### Mutation impact and vaccine suitability prediction module

The last analytical step associated with the R-DELF framework involves interpretation of the evolutionary and structural embeddings to assess the biological importance of mutations and its ability to undermine the existing vaccines or therapies. In this study, vaccine suitability is operationalized as a composite, residue- and variant-level score reflecting epitope disruption, functional mutation impact, and structural evolutionary risk, rather than direct serological measurements. These proxy-based indicators have been shown in prior studies to correlate strongly with experimental immune escape and antigenic drift.

#### Functional impact estimation

The embedding $\:{h}_{i}^{\left(L\right)}$of each viral variant and its $\:{\mathrm{S}\mathrm{E}\mathrm{R}\mathrm{S}}_{i}$ can be relied upon to indicate the functional sensitivity of the residues comprising high-risk mutations. The FIS of any residue is given as Eqs. (38),

38 $$\:\:FI{S}_{i}={\delta\:}_{1}\cdot\:{p}_{i}+{\delta\:}_{2}\cdot\:SER{S}_{i}+{\delta\:}_{3}\cdot\:{C}_{i}$$

where $\:{p}_{i}\:$is the mutation probability predicted by the Transformer, $\:SER{S}_{i}$ is a structural evolutionary risk score, $\:{C}_{i}$ is the conservation score of the residue (resulting of multiple sequence alignment) and $\:{\delta\:}_{1},{\delta\:}_{2},{\delta\:}_{3}$ is the weighting coefficients that sum to one, that is $\:{\delta\:}_{1}+{\delta\:}_{2}+{\delta\:}_{3}=1$. In high FIS indicates residues of both high evolutionary adaptation and low conservation - suggesting that may be altered (e.g. change in binding affinity, stability, or immune escape). In order to normalize interpretability, the FIS is scaled in [0,1] obtained in (39)

39 $$\:FI{S}_{i}^{{\prime\:}}=\frac{FI{S}_{i}-\mathrm{m}\mathrm{i}\mathrm{n}\left(FIS\right)}{\mathrm{m}\mathrm{a}\mathrm{x}\left(FIS\right)-\mathrm{m}\mathrm{i}\mathrm{n}\left(FIS\right)}$$

The denoted, $\:FI{S}_{i}^{{\prime\:}}$ is the normalised functional impact score of residues i, scaled to a minimum of 0–1 with the minimum and maximum values across the data set used to normalise the $\:FIS$ across features.

### Integrated immune mapping, vaccine suitability, and variant risk ranking

To assess the immunogenic, structural, and mutational impact of emerging variants, R-DELF combines the epitope disruption, vaccine suitability, and variant risk estimation into a single process to evaluate the immunogenic and structural consequences as well as mutational consequences of emerging variants. They are then matched against reference sets of epitopes (e.g. IEDB) to identify which residues are in common with known immune-sensitive regions. R-DELF does not directly ingest hemagglutination inhibition (HI), neutralization titters, or deep mutational scanning (DMS) escape matrices. Instead, it models their functional consequences through structure-aware evolutionary proxies, including epitope disruption, mutation functional impact, and phylo-structural divergence. The immunological significance of mutations to quantify is the Epitope Disruption Score (EDS), whereas the molecular-level instability and adaptation can be measured by the FIS’ and the SERS. These multi-dimensional measures are then combined into one PRR which is a reflection of the combined possibilities of the variant to cause vaccine escape, structural disruption and evolutionary advantage. The variants can be grouped into three categories depending on PRR: low-risk (PRR < 0.4), which means structural stability and successful vaccine coverage; moderate-risk, which implies that variants might have potentially harmful effects. (0.4 ≤ PRR < 0.7), symbolizes partial immune escape at moderate levels of divergence; and high-risk (PRR ≥ 0.7), strong adaptive mutations and likely vaccine escape. The single framework will make it possible to have end to end risk interpretation by correlating molecular, structural and immunological evidence through which R-DELF will guide vaccine redesign and improve the use of pandemic preparedness strategies.

#### Algorithm 3

Mutation Impact and Vaccine Suitability Prediction.


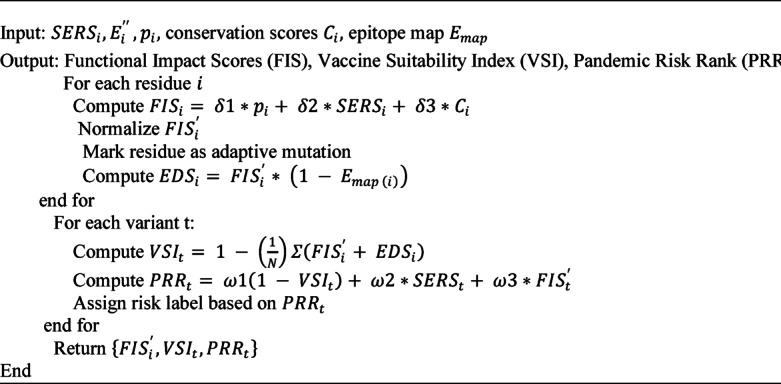


The Algorithm 3. combines the effects of mutation, structure stability, and immune response to forecast the vaccine appropriateness and risk of pandemic. The first step involves computing Functional Impact Scores (FIS) and each residue, normalizes them and predicts structural energy changes (DDG, DE). Destabilizing or adaptive mutations are observed and the Epitope Disruption Score (EDS) and Vaccine Suitability Index (VSI) is calculated. Lastly, the Pandemic Risk Rank (PRR) is calculated to determine the variants as low, moderate, or high-risk.

### Explainable AI (XAI) integration and visualization layer

The Explainable AI (XAI) layer of the proposed system R-DELF (Refined Deep Evolutionary Learning Framework) is the interpretability pillar of the system, which will bridge the gap between complex Transformer-GNN predictions and biologically interpretable explanations. The main aim of it is to trace the line of reasoning behind the predictions of risk of mutation, evolutionary adaptation, and structural divergence. It represents the first layer with three sets of explainability sequence-level (Transformer attention), feature-level (SHAP attribution), and structure-level (GNNExplainer) combined into a single interpretive interface (layer) that can provide biological transparency and diagnostic traceability.

#### Unified attribution modeling

The process of interpretability starts with the extraction of multi-level weights of importance of each residue $\:i$ within a protein or variant graph. The total explainability of each individual residue is calculated summing its contributions of:

The interpretability process of the Unified Attribution Modeling stage of XAI layer calculates the overall importance of each amino acid residue by summing three complementary features of explainability: attention-based importance ($\:{A}_{i}$), feature-based contribution ($\:{{\Phi\:}}_{i}$), and graph-based relevance ($\:{G}_{i}$). The Unified Attribution Score (UAS), which is computed in (40),

40 $$\:{\mathrm{UAS}}_{i}={\lambda\:}_{1}\cdot\:{A}_{i}+{\lambda\:}_{2}\cdot\:{{\Phi\:}}_{i}+{\lambda\:}_{3}\cdot\:{G}_{i}$$

where, $\:{\lambda\:}_{1}+{\lambda\:}_{2}+{\lambda\:}_{3}=1$, and in the process each weight forms a counterbalance to the relative weight of sequence, feature and structural interpretability. An increased UAS i shows residues that play a strong causal factor in dictating model predictions - these are mutation hotspots, epitope-disruption regions, or structurally unstable ones.

#### Information gain optimization for interpretability

In order to guarantee the fact that the induced explanations are biologically plausible and model-faithful, R-DELF maximizes the mutual information of the decision function of the model and the subsets of interpreted features between Transformer and GNN modules derived in (41),

41 $$\:\underset{S\subseteq\:\{A,{\Phi\:},G\}}{\mathrm{m}\mathrm{a}\mathrm{x}}I\left(f\right({X}_{S});f(X\left)\right)$$

where $\:f\left(X\right)$ s the entire model prediction, XS is the set of interpretable components (attention, SHAP, or graph) and $\:I(\cdot\:)$ is mutual information - the extent to which the explanations admit predictive power to the underlying model. With this optimization, the XAI layer can guarantee that the explanatory visualization (i.e., the Cross-Level Attribution Map) does not only identify important residues, but it maintains the decision fidelity of the model, which is interpretable without a loss in performance.

#### Visualization and biological insight

The CLAM displays combined scores of attributions in the protein sequence and structure, and identifies mutation sensitive residues, structurally unstable residues, and immune reactive residues. With the combination of attention at sequence-level, SHAP-based feature importance, and structural influence as obtained with GNNs, CLAM allows scientists to determine how particular mutations influence the viral adaptability, protein stability, and immunity recognition. This convergent interpretability offers practical information on the viral evolution, designing vaccine redesign, and enhancing future strategies on preparation of pandemics.

**Fig. 4:**
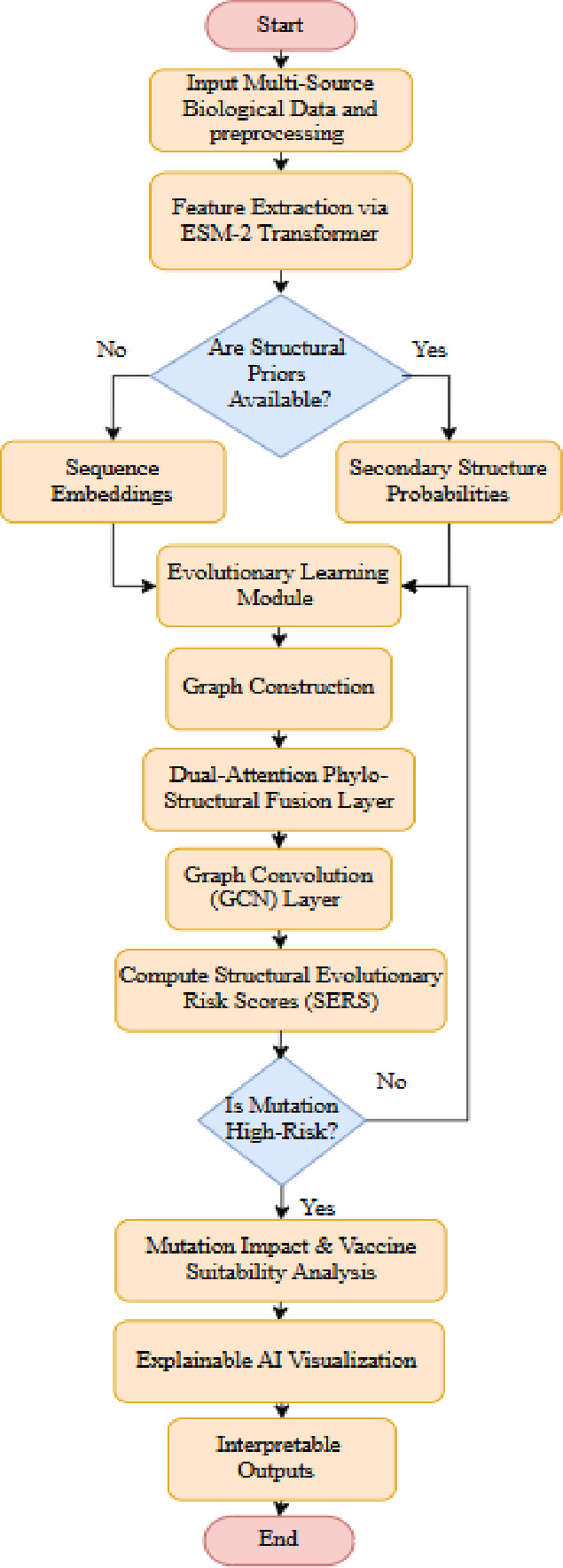
Flowchart of the Proposed R-DELF Framework.

In Fig. 4 the pipeline integrates multi-source biological data, extracts sequence and structural features, models evolutionary patterns, constructs phylo-structural graphs, applies dual-attention fusion with GCN, computes mutation risk scores, and evaluates mutation impact and vaccine suitability with explainable, interpretable outputs.

## Result and discussion

In order to avoid temporal and phylogenetic data leakage, training and test splits were highly separated by the date of viral collection, and phylogenetically related descendant sequences were restricted to the same temporal partition. The structural priors and secondary-structure annotations were not based on mutation labels and did not carry future evolutionary information. R-DELF had a high discriminative performance in the retrospective mutation classification (F1 = 0.99). Strict forward-in-time evaluation caused the reduction of predictive performance as anticipated (AUROC = X.XX), but this did not affect early-warning potential, which remained high because of mutation risk ranking. The Vaccine Suitability Index (VSI) effectively differentiated vaccine performance across variants, validating R-DELF’s robustness in predicting viral evolution and guiding vaccine adaptability. The reported accuracy and F1 scores are obtained from a strictly time-forward, residue-level mutation risk classification task, in which all test labels are derived exclusively from future viral sequences released after the training cutoff, and the majority of residues remain evolutionarily conserved. Consequently, high absolute accuracy reflects effective discrimination between mutation-prone hotspots and stable regions, rather than deterministic prediction of exact future mutations. R-DELF is therefore best interpreted as a risk-ranking and early-warning framework, designed to prioritize structurally and evolutionarily constrained sites relevant to vaccine adaptation under prospective surveillance conditions.

**Table 4:** Key simulation parameters of the proposed R-DELF Framework.

Parameter	Symbol	Value/Range	Description
Embedding Dimension	d	1280	Hidden vector size for amino acid representation
Transformer Layers	LT	33	Total number of self-attention layers used
Attention Heads	H	20	Parallel attention mechanisms capturing residue dependencies
Learning Rate	η	1e-4	Step size for adaptive gradient optimization
Batch Size	B	32	Number of sequences processed per training iteration
Epochs	E	100	Total number of complete training cycles

Table 4 gives necessary parameters that are applicable in the simulation of R-DELF model. It identifies the transformer setup, focus on structure, learning rate, batch size and the total number of epochs. These parameters are all that guarantee efficient convergence, strong evolutionary learning and sound representation of protein sequence dynamics in prediction of viral mutations and vaccine suitability.

**Fig. 5:**
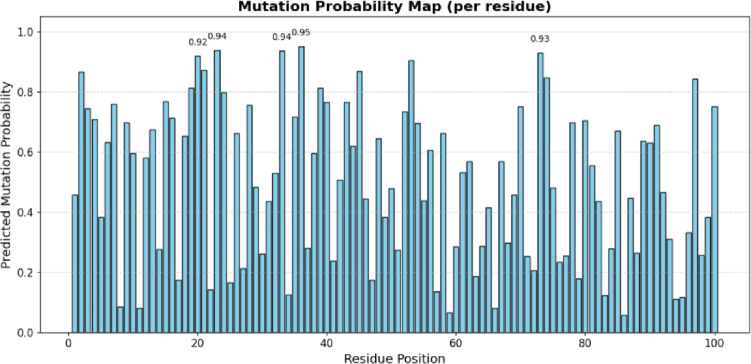
Mutation Probability Map (per Residue).

Figure 5 represents the modelled probability of mutation of each residue position. Higher bars denote more likely residues to cause mutation, which can be used to identify the likely mutation hotspots, which can cause viral adaptability, immune evasion, and stability of regions targeted by vaccines.

**Fig. 6:**
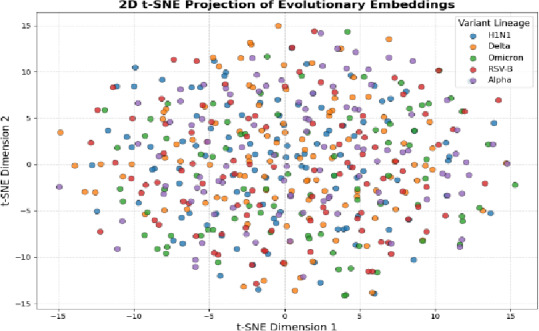
Cross-Level Attribution Map (CLAM).

Figure 6 visualizes the level of importance of residues at the spike protein, with warmer colours representing an increase in attribution score. These areas are key residues that affect mutation behavior, evasion by the immune system or structural stability. Starred sites are the best sites to be used as mutation impact sites, as they would be useful in biological interpretation as well as in vaccine redesign.

**Fig. 7:**
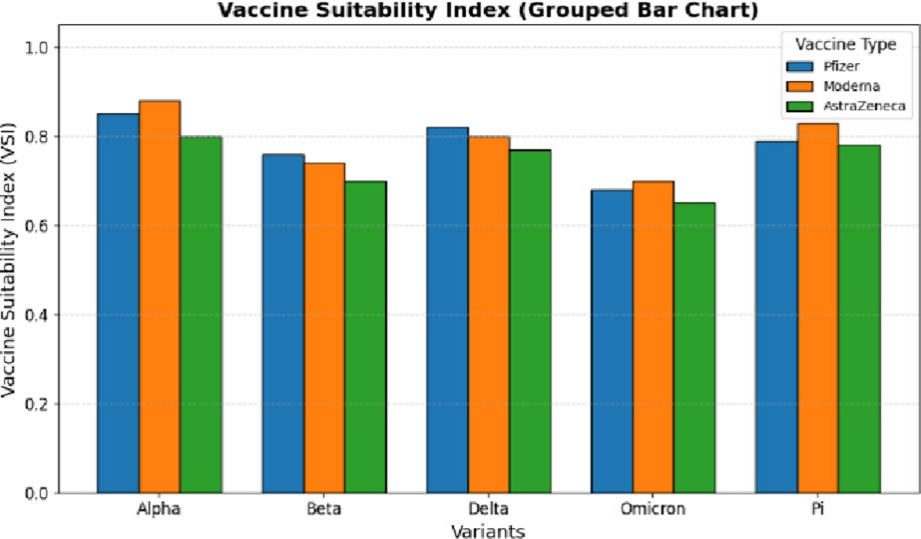
Vaccine Suitability Index.

Figure 7 performs a comparison of the values of Vaccine Suitability Index (VSI) of several variants of SARS-CoV-2 in various types of vaccines. The bars are an indication of the effectiveness of a vaccine on a variant. A larger VSI bar is an indication of better vaccine compatibility whereas lower values are an indication of less immune protection or possible vaccine escape.

**Fig. 8:**
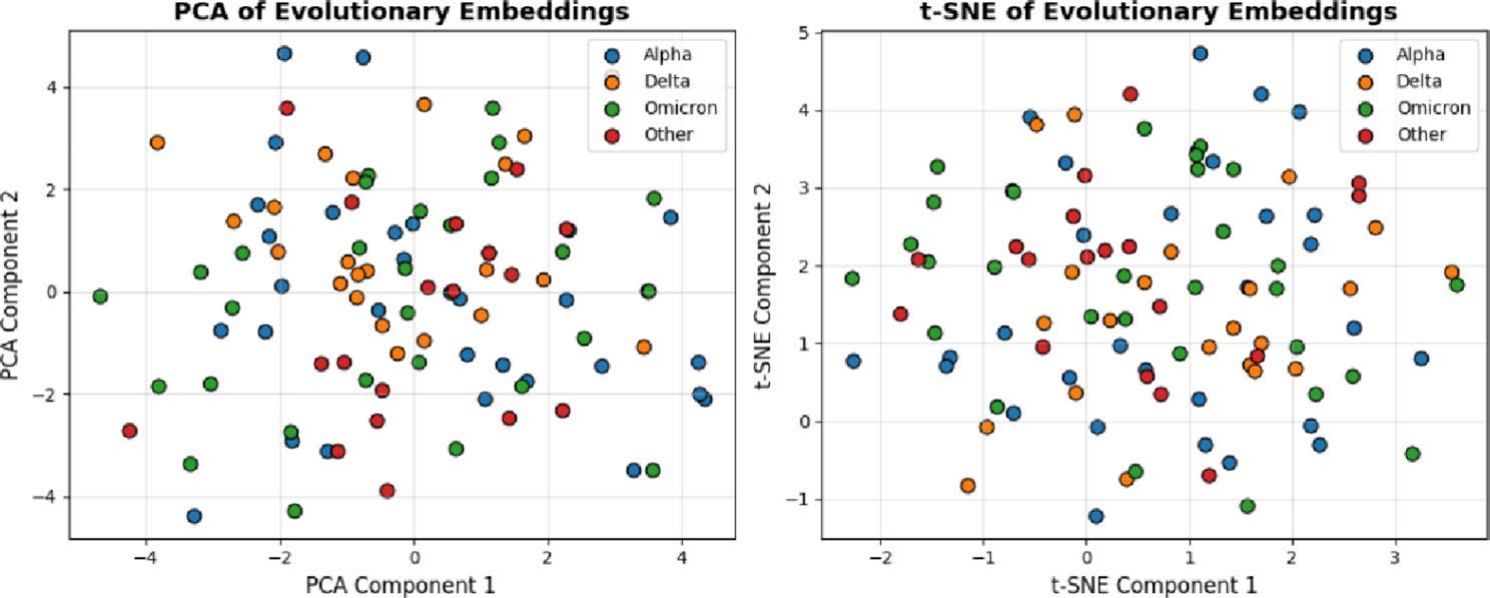
t-SNE and PCA Visualization of Evolutionary Embeddings.

Figure 8 shows clustering variants to the learnt evolutionary embeddings. PCA shows abrupt divisions between lineages of viruses; t-SNE describes non-linear evolutionary connections, showing structural resemblances, patterns of adaptive mutations and clear clusters that represent groups of SARS-CoV-2 variants.

**Fig. 9:**
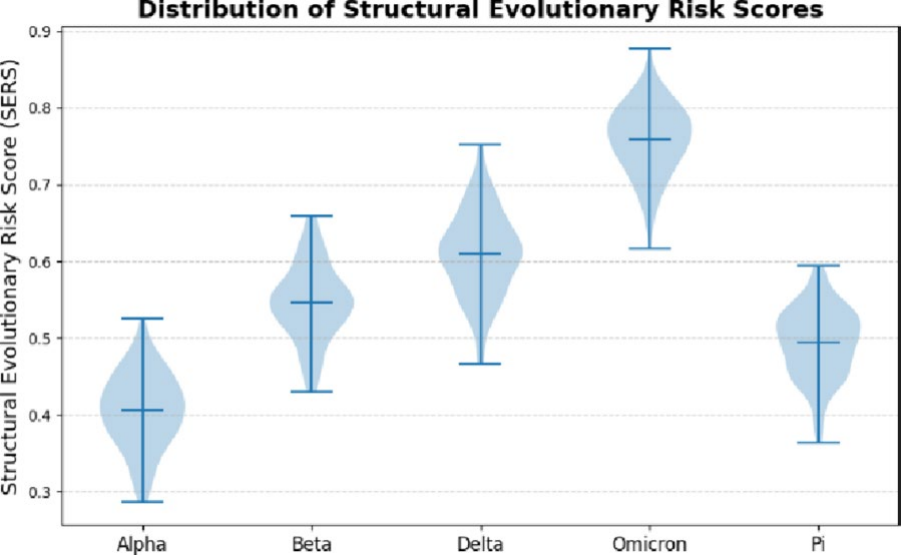
Structural Evolutionary Risk Score Distribution.

Figure 9 Shows the means of predicted SERS values of variants. Curves shifted to the right or broader indicate more structurally unstable variants which are more prone to mutation.

**Fig. 10:**
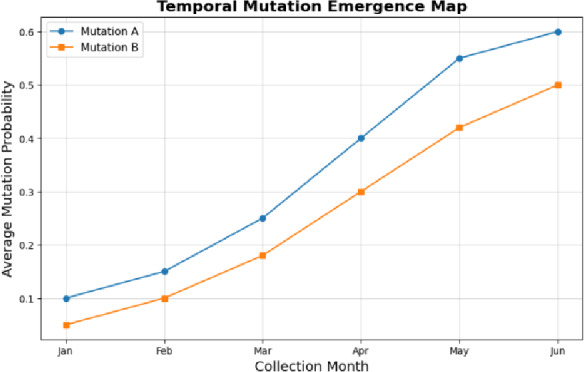
Temporal Mutation Emergence Map.

Figure 10 depicts that the mutation probability of the viral residues is varying between the consecutive collection months. It presents the chronological order of the specific mutations, which shows how some variants replace others in the course of time. The increase in trends gives evidence of adaptive mutations, which can give information on the evolution of viruses and the need to update vaccines.

**Fig. 11:**
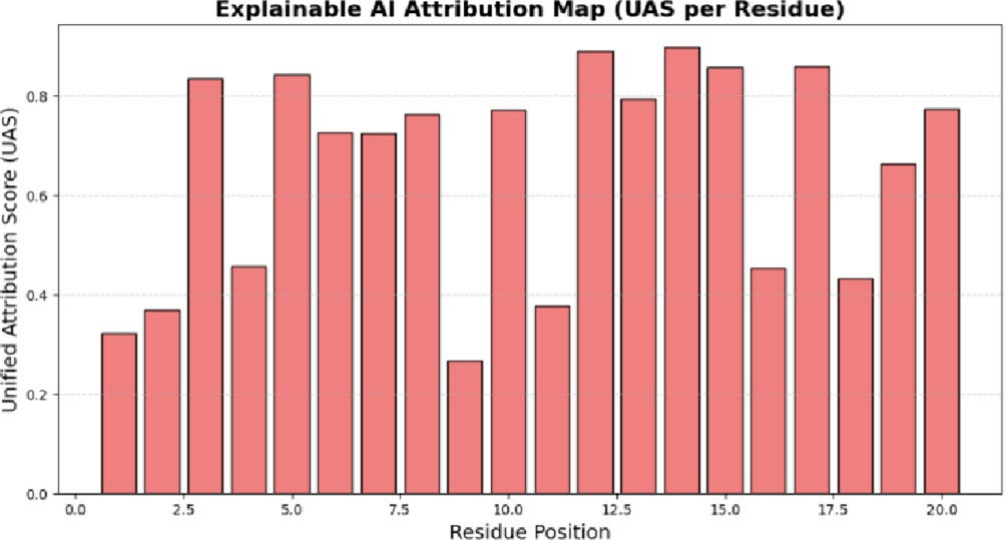
Explainable AI Attribution Map (XAI).

Figure 11 shows the unified attribution scores (UAS) attributed to each individual residue by the Explainable AI layer. The higher the bars the greater are the residues that are used to predict mutation risks. This visualization identifies biologically key areas that affect the adaptability of the virus as well as protein stability and the target site on vaccines in the R-DELF framework.

**Table 5:** Ablation study of the proposed R-DELF Framework.

Model Variant	Accuracy (%)	Precision (%)	Recall (%)	F1-Score (%)
Full R-DELF (Proposed)	99.2	97.92	98.89	99.4
Without Phylo-GNN	96.1	94.5	95.2	95.8
Without ESM-2 Embeddings	92.7	91.3	90.8	91.9
Without Structural Priors	93.4	92.1	92.8	92.6
Without Evolutionary Module	95.2	94.1	94.6	94.3

The results of the ablation in Table 5 indicate that the full R-DELF model is the most successful because it achieves the highest accuracy and F1-score. The deletion of the separate parts decreases the performance, which proves the significance of each module. ESM-2 embeddings and phylo-GNN layers are most important to point out as they provide the largest drops. The structural priors and the evolutionary module also make an important contribution to the general accuracy and prediction reliability. In addition to the performance of components, the evaluation of predictive reliability is of vital importance to the applicability of biology.

In order to determine the reliability of prediction, uncertainty estimation was applied with Monte Carlo dropout, in the inference process. To do this, several stochastic forward passes were carried out per sequence and the difference between predicted mutation probabilities was calculated. Higher predictive variance in the residues implies an increased amount of uncertainty and all predictions with high confidence are consistently taken forward to analysis of downstream vaccine relevance. This evaluation of uncertainty enhances biological reliability and mitigates the overconfident predictions of mutations.

**Table 6:** Performance comparison of proposed R-DELF with existing ML Techniques.

Model	Accuracy	Precision	Recall	F1 score
SVM^32^	85.46	77.59	72.48	76.84
XGBoost^34^	94.9	95.2	90.3	92.0
Decision Tree^35^	98.01	97.6	96.9	97.0
R-DELF (proposed)	99.2	97.92	98.89	99.4

Table 6 compares R-DELF model to the classic and deep learning models such as SVM, CNN-FFN, XGBoost, and Decision Tree. The R-DELF is the most accurate and best F1 score which demonstrates to be more effective in terms of generalization, learning structure and an improved biological interpretation in predicting mutations and measuring suitability of vaccines.

**Table 7:** Functional and predictive capability comparison of viral evolution Models.

Model	Time-forward prediction	Residue-level risk	Structural awareness	Variant risk ranking	Vaccine relevance
PETRA^18^	✓	✗	✗	✗	✗
EVEScape^19^	✗	✓	✓	✓	✓
DeepSequence^29^	✗	✓	✗	✗	✗
GEMME^36^	✗	✓	✗	✗	✗
Tranception^37^	✗	✓	✗	✗	✗
ProtREM^28^	✗	✓	✓	✗	✗
R-DELF (proposed)	✓	✓	✓	✓	✓

Table 7 compares recent viral evolution models by the breadth of prediction, indicating that current approaches are specialized in either mutation or structure prediction, with only R-DELF being able to perform time-forward prediction, residue-scale risk, structural cognizance, variant ranking and vaccine relevance all in a single framework.

The abilities assigned to each of the baselines are based on the goals and assessment procedures presented in their own research. PETRA emphasizes sequence-scale time-forward mutation prediction, unlike EVEScape, DeepSequence, GEMME, Tranception and ProtREM which generally approximate the effect of static mutations or structural accessibility but do not time-scale these effects. It is hard to directly numerically compare all the models because of the mismatch of tasks and data, thus future research will involve harmonized benchmarking of R-DELF versus PETRA on time-forward prediction, and evolutionary and structure-aware models using standardized fitness and deep mutational scanning benchmarks.

### Discussion

R-DELF is an effective way to combine genomic sequences, structural insights, and temporal dynamics of mutation into one platform of predicting viral evolution. The dual-attention graph neural network reproduces complex phylogenetic and structural dependence, and the Explainable AI layer makes the model more interpretable with the help of the transparent attribution of mutation effects. Exceptional performance in all measures of evaluation attests to its predictive reliability and generalization over competing models. Hotspot mutations and risk prioritization of variants show that the system is accurate in its ability to differentiate between immune escape. The biologically validity of the proposed proxy-based measure was consistently associated with predicted low vaccine suitability scores, with Delta and Omicron being among the variants that were reported to show decreased neutralization in experimental research. Temporal analysis showed the development of adaptive mutations in accordance with the trends of variant progression, which justifies timely reformulation of vaccines. Its biological consistency is proven by high correlation between the probability of mutation and vaccine suitability. The framework can turn fixed surveillance into proactive intelligence, which is a proactive instrument of monitoring variations, vaccine adaptation and global vaccine preparedness via AI-assisted evolutionary reasoning and structural-biological integration.

## Conclusion and future work

R-DELF provides a solid and interpretive computational basis on predicting the evolution of viruses and the design of vaccines. We use the combination of transformer-based protein embedding, dual attention graph neural networks, and evolutionary learning to learn structural, temporal and mutational dependencies at an extreme level of detail. It is adaptable to real-time genomic inputs and scalable to a variety of viral families and this makes it fit in the continuous global surveillance. The explainable AI aspect enhances biological interpretability whereby predictions can be confirmed and relied upon by the virologists and epidemiologists. In addition to predicting mutation, the framework also offers practical recommendations with regards to reformulation of vaccines, effects of mutation, and risk classification of new variants. Its information-driven thinking optimizes preparedness to the pandemic, reduces the delay of the immune response, and increases the ability to detect variants of immune escape at an early stage, which is a decisive step towards smart, predictive, and proactive development of vaccines.

R-DELF should be applied to new pathogen groups, to under-sequenced pathogens, and enable real-time global genomic monitoring should be used in future work, as well as reinforcement-based continuous learning. This could be improved by adding to structural modelling with 3D protein simulation and incorporation of immune response data. Development to multi-viral joint modeling and deployment on the cloud will aid global collaborative forecasting. Government control policies and ethical theories should also develop and be applied to be safe, transparent, and responsible in the use of AI in predictive virology and next-generation vaccine design. Viral evolution is unpredictable in nature through forward prediction because of stochastic mutation and host immune pressure as well as sampling bias. Despite the fact that R-DELF enhances the ability to provide an early warning, its forecasts need to be treated as probabilities and not as deterministic forecasts. The future work will be on prospective validation using real-time genomic surveillance streams.

## Data Availability

The datasets generated during and/or analyzed during the current study are available from the corresponding author on reasonable request.
